# Supplementary material for: An Egocentric Network Contact Tracing Experiment: Testing Different Procedures to Elicit Contacts and Places
Source: Int J Environ Res Public Health. 2021 Feb 4;18(4):1466. doi: 10.3390/ijerph18041466 (PMC7916034; doi:10.3390/ijerph18041466)
Supplement: Supplementary file 1 [file ijerph-18-01466-s001.pdf]

Questionnaire

Race

Which option best describes your race?

(a) American Indian or Alaska Native
(b) Asian
(c) Black or African American
(d) Hispanic or Latino Origin
(e) Native Hawaiian or Other Pacific Islander
(f) White
(g) Other

Gender

Which option best describes your gender?

(a) man
(b) woman
(c) non-binary
(d) prefer not to disclose
(e) prefer to self-describe

Mood: (Rholes et al. *[26]; Cronbach's alpha = 0.91)*

(a) I am generally in an elated mood.
(b) I am generally in a cheerful mood.
(c) I am generally in a happy mood.
(d) I am generally in a good mood.
(e) I am generally in a sad mood.
(f) I am generally in a discouraged mood.
(g) I am generally in a depressed mood.
(h) I am generally in a bad mood.

The answer categories were

(1) Strongly disagree
(2) Disagree
(3) Neither agree nor disagree
(4) Agree
(5) Strongly agree

Well-being: (Shea et al. *[13]; Cronbach's alpha = 0.86)*

(a) How satisfied are you with your standard of living?
(b) How satisfied are your with your health?
(c) How satisfied are you with what you are achieving in life?
(d) How satisfied are you with your personal relationships?
(e) How satisfied are you with how safe you feel?
(f) How satisfied are you with feeling part of your community?
(g) How satisfied are you with your future security?
(h) How satisfied are you with the amount of time you have to do the things that you like doing?

The answer categories were

(1) Strongly dissatisfied
(2) Dissatisfied
(3) Neither satisfied nor dissatisfied
(4) Satisfied

(5) Strongly satisfied

Physical functioning: (Ross and Mirowsky [27]; Cronbach's alpha = 0.81)

Do you have any trouble or difficulties

(a) walking?
(b) using stairs or inclines?
(c) standing or sitting for long periods?
(d) using your fingers to grasp or handle?
(e) lifting or carrying something as heavy as 10 pounds?

The answer categories were

(1) No
(2) Yes, some
(3) Yes, a great deal

Mask efficacy

Do you think wearing a mask helps to reduce the spread of COVID-19?

The answer categories were

(1) No, it increases the spread
(2) No, it does nothing
(3) Not sure
(4) Yes, some
(5) Yes, a lot

COVID: Worry

How worried are you about getting COVID-19?

The answer categories were

(1) Not worried at all
(2) Somewhat worried
(3) A little worried
(4) Very worried

COVID: Seriousness

On a scale of 1–10, how serious of a public health threat is COVID-19?

The answer categories were 1 = being no threat and 10 = being very serious
